# Donor dependency and pandemic readiness: insights from the 2026 Ebola Bundibugyo outbreak in the East African community

**DOI:** 10.3389/fpubh.2026.1891266

**Published:** 2026-07-15

**Authors:** John Mugisha, Julius Niyibizi, Emmanuel Nizeyimana, Munawar Harun Koray

**Affiliations:** 1 Rwanda Implementation Science & Health Systems Lab (RISHS Lab), Kigali, Rwanda; 2 Southern Medical University, Guangzhou, China

**Keywords:** Bundibugyo virus disease, cross-border collaboration and surveillance, Democratic Republic of the Congo, donor dependency, East African community, health security financing, outbreak research infrastructure

## Abstract

The 2026 Bundibugyo virus disease (BVD) outbreak has exposed persistent weaknesses in health-security preparedness across the East African Community (EAC). Although regional preparedness has improved, major gaps remain in early detection, cross-border collaboration and surveillance, laboratory capacity, infection prevention and control, and community engagement. These vulnerabilities have been intensified by declining external health-security funding since 2025, incomplete implementation of domestic health-financing commitments, and the fragility of systems that were built with substantial donor support but not fully institutionalized. Drawing on the current outbreak and the extensive experience with Ebola virus disease in the Democratic Republic of the Congo (DRC), this Perspective argues that the outbreak should be understood not only as an infectious-disease emergency but also as a stress test for donor-supported preparedness systems. It calls for phased transitions from donor-supported functions toward sustained domestic and regional financing, permanent cross-border surveillance, strengthened peripheral laboratory capacity, institutionalized community-based preparedness, and outbreak-ready clinical research platforms.

## 1 Introduction

The East African Community (EAC) is home to more than 300 million people across active infectious-disease corridors where cross-border movement, zoonotic spillover risk, and uneven health-system capacity increase vulnerability to viral hemorrhagic fevers (1–3). Ebola virus disease (EVD) is not new in this region. Since 2000, outbreaks and cross-border importations have involved *Orthoebolaviruses*, including *Orthoebolavirus zairense*, Orthoebolavirus sudanense, and Orthoebolavirus bundibugyoense (4–7). Previous responses have produced temporary gains, but these have often been difficult to sustain once emergency resources decline (8, 9).

The current outbreak is unfolding in a changed health-financing environment. Since 2025, reductions and realignments in U.S.-supported health security programs have raised concerns about surveillance, laboratory, rapid-response, and workforce continuity in EAC settings (10–12). From 2014 to the present, the Global Health Security Agenda (GHSA) and related bilateral investments created real capacity in Uganda, Kenya, Tanzania, Rwanda, and other EAC countries (13–15). However, many of these capacities remain insufficiently embedded in domestic financing and routine public health ownership.

This Perspective argues that the Bundibugyo outbreak is both a health emergency and a stress test for donor-supported preparedness systems. The problem is not donor support itself, but abrupt or unplanned withdrawal before local and regional systems can absorb critical functions. Sustainable preparedness requires a gradual transition that protects donor-supported gains while building domestic financing, regional pooling, workforce planning, and routine public health systems.

## 2 The 2026 Ebola Bundibugyo outbreak: epidemiological context

The Bundibugyo virus (BDBV), first identified during the 2007–2008 outbreak in Uganda, is now recognized as Orthoebolavirus Bundibugyoense (16). WHO describes the case fatality ratio in past BDBV outbreaks as generally 30% to 50%, although estimates vary by case ascertainment, supportive care, and context (17–19). The approved rVSV-ZEBOV vaccine (Ervebo), effective against Orthoebolavirus zairense, does not target BDBV, and no licensed BDBV-specific vaccine or therapeutic exists (20). This gap alters the outbreak’s risk calculus.

The Democratic Republic of the Congo (DRC) Ministry of Health formally declared the current outbreak on 15 May 2026, after the WHO was alerted on 5 May to a high-mortality illness in Mongbwalu Health Zone, Ituri Province, and INRB Kinshasa confirmed BDBV in samples from Rwampara Health Zone (19). The early epicenters included Mongbwalu, Rwampara, and Bunia health zones, followed by spreading across Ituri and into North and South Kivu (21). Cross-border spread into Uganda likely occurred along established population-movement routes. Uganda’s first confirmed imported case was detected in Kampala in a Congolese man who had traveled from the DRC and died on 14 May 2026 (19).

By 24 June 2026, WHO reported 1,000 confirmed cases and 254 deaths in the DRC across 33 health zones, while WHO and CDC reported that Uganda had 20 cases and two deaths by 24 June (21, 22). CDC reported that by 22 June the DRC had confirmed more than 1,094 cases, and 277 deaths making this the second-largest Ebola outbreak on record (22). This rapid increase highlights common risks that worsen outbreaks, such as late detection, lack of monitoring, community pushback, and ongoing gaps in IHR capacity, which were also seen in West Africa and during the 2018–2020 outbreak in North Kivu and Ituri in the DRC (23, 24).

## 3 EAC preparedness architecture and Lessons from DRC experience: evidence of Progress and its real limits

Uganda has built institutional memory in its Ebola response since the 2000 Gulu outbreak. Post-outbreak reforms strengthened rapid-response systems; the Uganda Virus Research Institute (UVRI) in Entebbe provides confirmatory filovirus diagnostics; and Joint External Evaluations have documented improvements in laboratory systems and emergency operations center functionality (7, 14, 25). These gains are important, but they should be interpreted alongside the DRC’s broader experience as an EAC member state and the country most affected by Ebola virus disease globally.

The DRC experience is especially relevant because the country has experienced more Ebola outbreaks than any other country and has confronted outbreaks caused by different ebolavirus species in forested, urban, cross-border, and conflict-affected settings. The 2018–2020 North Kivu and Ituri outbreak occurred amid armed conflict, population displacement, attacks on health facilities, misinformation, and distrust, yet it also generated operational lessons in community engagement, vaccination logistics, contact tracing under insecurity, and coordination between national authorities and international partners (23, 26).

Repeated outbreaks in Equateur Province also offer lessons for decentralized preparedness. During the 2020 Equateur outbreak, decentralized treatment approaches and the use of existing health facilities as decentralized treatment centers were explored to improve access and reduce delays in care-seeking and diagnosis (27). Decentralization relied on pre-identified isolation rooms in district hospitals and rotating clinical teams rather than fixed Ebola treatment units. For the EAC, this experience suggests that preparedness should not be concentrated only in national capitals or reference hospitals; district and peripheral systems need pre-positioned capabilities, referral pathways, and safe isolation capacity.

The evolution of the DRC’s National Institute for Biomedical Research (INRB) illustrates the positive side of donor-supported capacity building. INRB has become a central national institution for outbreak confirmation, genomic characterization, and laboratory coordination. During the North Kivu response, in-country whole-genome sequencing capacity was established at INRB, enabling genomic evidence to support outbreak investigations, transmission chains, and operational decision-making (28). This required sustained financing for reagents, equipment maintenance, and trained bioinformatics staff between outbreaks, not just during emergencies. This experience demonstrates that external support can strengthen sovereign capacity when investments are embedded in national institutions.

The DRC outbreaks have also helped the world get ready for Ebola by conducting clinical research and following up with survivors. The PALM trial, which took place during the 2018–2020 North Kivu and Ituri outbreak, tested new drugs during an active emergency and helped show that antibody-based treatment works for Zaire ebolavirus disease (29). Survivor cohorts and longitudinal research programs in the DRC have further shown the importance of follow-up for sequelae, viral persistence, relapse risk, stigma, and reintegration (30). These are not peripheral achievements; they are core components of pandemic readiness.

At the regional level, Africa CDC, the East Africa Integrated Disease Surveillance Network/African Field Epidemiology Network (EAIDSNet/AFENET), and WHO AFRO’s Integrated Disease Surveillance and Response framework provide platforms for surveillance support, data sharing, emergency coordination, and district-level alert and response (3, 31, 32). The EAC Health Policy Framework and Global Outbreak Alert and Response Network also support cross-border notification, joint response, and surge capacity (33). One Health frameworks have expanded in several EAC countries, but implementation remains uneven and continues to lag in many border and forest-interface settings (34, 35).

## 4 Persistent gaps and structural barriers in EAC preparedness

### 4.1 Early detection and alert systems

Reported delays between symptom onset, alert, specimen transport, and laboratory confirmation remain a major vulnerability. At district and community levels, alert systems are fragmented, and rural or border-area clinicians may not suspect Ebola virus disease early because malaria, typhoid, and other febrile illnesses present similarly (17, 36). Community-based and One Health surveillance networks exist but remain unevenly resourced between outbreaks, while GHSA-supported surveillance officers are not always retained or supervised after external project support declines (34, 35, 37).

### 4.2 Border surveillance and cross-border coordination

Formal points of entry have screening processes and staff, but many informal crossings through footpaths, rivers, and forests bypass routine surveillance. Joint external evaluations and regional health security assessments conducted in East and Central Africa have repeatedly highlighted weaknesses in surveillance, cross-border collaboration, and preparedness (24, 38). The 2026 outbreak illustrates this vulnerability. Uganda’s first confirmed imported case was detected in Kampala rather than at a formal point of entry.

Cross-border epidemiological data sharing between Uganda and the DRC has functioned sporadically. Varying case definitions hamper joint contact tracing, inadequate secure communication between teams and challenges posed by non-state armed groups in eastern DRC, all of which can undermine recognition of public health authorities (23, 39). Protocols alone cannot address these challenges. They require sustained, funded, staffed coordination mechanisms that can continue even when donor funding is reduced or reconfigured.

### 4.3 Laboratory capacity at the peripheral level

Confirmatory testing for BDBV still requires specimen transport to national reference laboratories, such as UVRI in Entebbe or INRB in Kinshasa, or, in some cases, to international reference institutions. The operational implications are substantial: longer collection-to-result times can delay isolation and contact tracing at a critical point in an outbreak (40). Point-of-care filovirus diagnostics, such as GeneXpert systems tested in the field, are not widely available in the EAC’s peripheral laboratory network (41–43). The DRC experience demonstrates that investments in in-country sequencing and laboratory systems can shorten the distance between outbreak investigation, confirmation, and operational decision-making, but only if equipment, reagents, biosafety systems, data pipelines, and trained staff are sustained between outbreaks (28).

### 4.4 Infection prevention and control in peripheral health facilities

Personal protective equipment (PPE) and infection-prevention commodities are often insufficient during outbreaks, forcing emergency procurement rather than planned stockpiling (44, 45). Frontline health care workers are often the first point of contact for patients with Ebola virus disease, yet they may lack adequate protection, as seen in the 2007 Bundibugyo outbreak, the 2022 Sudan outbreak, and the ongoing BDBV outbreak.

Infection Prevention and Control (IPC) training is usually conducted following outbreaks rather than through regular programming due to constraints that hinder sustained IPC capacity building in fragile settings (46, 47). Attributing the erosion of preparedness solely to staff transfers or skill loss overlooks the more important structural issue of inadequate funding for sustainability: routine refresher training, supportive supervision, mentorship, and train-the-trainer models are rarely financed long enough to ensure the long-term availability of qualified IPC personnel in peripheral facilities.

### 4.5 Community engagement and the trust deficit

The most difficult preparedness gaps are not only technical; they also concern community trust. A study carried out during the 2018–2020 North Kivu and Ituri outbreak in the DRC found that trust in health authorities was associated with self-reporting of illness and participation in contact tracing and treatment (23). Even when technical tools are available, surveillance systems may fail in areas where distrust stems from state neglect, violence, and misinformation.

Community health workers in western Uganda have reported that some community members may hide sick relatives from contact-tracing teams, partly because risk communication is perceived as surveillance rather than support (48, 49). The bigger issue is that community engagement is seen as a limited-scope outbreak response rather than an ongoing role in the health system. Trust-building efforts involve an ongoing, continuous presence, remuneration, and accountability, which are often overlooked during non-outbreak periods (37, 50).

## 5 The financing fracture: what U.S. Funding disruptions actually mean

The U.S. has long been a major financier and technical partner for global health security in EAC states, especially surveillance, laboratory systems, rapid-response training, and IPC (24, 51, 52). Since 2025, disruptions to U.S.-supported programs have raised concerns about the continuity of preparedness functions and broader health-system supports, including workforce posts, supply chains, information systems, and supervisory structures. EAC member states have also received assistance from the European Union, the United Kingdom, France, CEPI, the Wellcome Trust, the World Bank, WHO, Africa CDC, and humanitarian organizations, though U.S. support has historically been particularly important given its scale, technical depth, and role in surveillance, laboratory strengthening, and workforce development (10, 53, 54).

Nor should donor-supported systems be portrayed solely as liabilities. DRC experience shows that donor investments, when aligned with national leadership, can strengthen surveillance networks, laboratory and genomic infrastructure, emergency operations, clinical research capacity, vaccine deployment, therapeutics evaluation, and workforce training (20, 23, 29, 55). The vulnerability is the absence of a predictable transition from externally supported emergency platforms to domestically financed, regionally coordinated, and routinely maintained public health systems (56–58). The policy goal should therefore be a time-bound transition compact, not permanent aid dependence or abrupt donor exit.

## 6 Action: recommendations for sustainable EAC health security

Although similar recommendations have appeared in previous outbreak evaluations, the current financing and preparedness crisis makes further delay increasingly difficult to justify.

### 6.1 Institutionalize domestic financing for health security

EAC member states should allocate preparedness funding at scale beginning in the next budget cycle. Like many African countries, EAC member states remain far from achieving the Abuja Declaration target on health financing and continue to rely heavily on donor support (59–61). Domestic financing commitments should be tied to specific, verifiable outputs: funded surveillance and IPC positions, maintained PPE and diagnostic stockpiles, functioning district laboratory referral systems, pre-positioned cross-border response funds, and annual simulation exercises. Joint External Evaluation (JEE) scores provide a usable baseline, and the EAC Secretariat Health Policy Framework offers the political architecture (24, 62). What has often been absent is budget action that matches these commitments.

### 6.2 Make cross-border surveillance a standing function, not an emergency activation

The EAC Health Protocol and the EAIDSNet framework (3, 62) contain mechanisms for joint cross-border surveillance. These systems must operate continuously rather than only following the declaration of an outbreak: health stations permanently staffed at critical crossing points, continuous epidemiological information exchange with the DRC, joint contact-tracing protocols, harmonized case definitions, shared risk communication materials, and year-round community-based surveillance with compensation for effort.

The Go.Data platform (63) offers a standardized, WHO-supported contact tracing and case management tool that both Uganda and DRC have used in prior outbreaks. Interoperability between country instances must be agreed upon, tested, and funded before future cross-border events, which requires resources and political agreement.

### 6.3 Build community health infrastructure as a permanent system

Communities with pre-existing trust relationships with health systems are more likely to report cases early, cooperate more with contact tracing, and accept referrals (48, 49). Trust requires permanent employment or reliable compensation for community health workers, not short-term mobilization only during epidemics. Continuous risk communication and community engagement should be co-designed with communities and routinely monitored. In DRC’s conflict-affected zones, this work must account for danger, skepticism, and the need to engage religious leaders, market traders, cross-border networks, and other trusted intermediaries (23, 50).

### 6.4 Integrate laboratory capacity into health system planning

GeneXpert equipment used for TB and HIV screening in the EAC should, where technically feasible, be expanded to include validated tests for filoviruses. In the event of an outbreak, trained personnel, appropriate biosafety measures, reliable cold-chain logistics, sample referral systems, quality assurance, and data linkage to surveillance platforms are critical. These technologies will matter only if they are embedded in staffed, financed, and quality-assured laboratory systems. The DRC’s INRB-supported genomic surveillance model shows that national laboratory leadership can generate operationally useful data, but this requires stable financing for reagents, maintenance, staff retention, and data governance (28).

### 6.5 Restructure regional response financing

The Africa Solidarity Trust Fund for Public Health Emergencies was created to reduce reliance on *ad hoc* emergency appeals. Member-state contributions have not been sufficient to make the fund a viable first-responder mechanism. A new contribution model that varies with GDP, including clear responsibilities for contribution drawdown and a regionalized procurement scheme for strategic PPE and diagnostic reserves, would reduce the response community’s reliance on emergency donor appeals (32). Such regional financing could fit into the WHO Health Emergency Preparedness, Response, and Resilience (HEPR) Framework (64). These mechanisms already exist, but their funding remains insufficient to function as reliable first-response instruments.

### 6.6 Create a phased transition compact with donors

EAC member states, Africa CDC, WHO, and major donors should agree on phased transition plans for surveillance, laboratory quality management, emergency operations, and IPC programs. These plans should specify which functions will remain externally supported, which will be co-financed, which will be absorbed into national budgets, and by when. Transition indicators should include funded posts, domestic procurement lines, training-of-trainers coverage, laboratory maintenance contracts, and operational readiness exercises. This approach would mitigate the risk that donor withdrawal undermines core health-system functions before domestic and regional capacities are firmly established.

## 7 Evaluation and scaling up: what accountability actually requires

Unmeasured improvements in preparedness will not be sustained. JEE scores provide a useful multi-domain baseline but are conducted infrequently and rely partly on self-reporting by the assessed country (24, 65). Complementing JEEs with annual after-action reviews tied to real events, including this outbreak, and with independent after-action verification, would create stronger accountability loops.

Regional-level evaluation mechanisms need to track cross-border surveillance functions, not just national-level capacities. A joint EAC framework for epidemiological surveillance performance indicators, one that measures alert-to-response time, cross-border notification timeliness, and contact tracing coverage at the interface zones, would provide the EAC Secretariat and Africa CDC the operational data needed to identify where the system breakdowns occur (38).

Scaling up preparedness requires explicit recognition of where existing systems have underperformed. Although ring vaccination was highly important in responses to *Orthoebolavirus zairense*, the absence of a licensed BDBV-specific vaccine limits the range of available biomedical countermeasures. Rapid development, evaluation, and accessibility of vaccines and therapeutics for BDBV are essential for EAC countries. The DRC experience with ring vaccination, the PALM therapeutics trial, and survivor follow-up shows that research and response can be integrated during active outbreaks when ethical review, trial governance, clinical care platforms, data systems, and community engagement are prepared in advance (20, 29, 30).

## 8 Discussion and conclusion

The recommendations outlined above may not be fully implemented before this outbreak is over. Political momentum for domestic health-security financing often rises during the peak of an outbreak and declines once the immediate emergency subsides. Similar patterns were observed across multiple EVD outbreaks in Uganda and the DRC, including the 2018–2020 North Kivu and Ituri outbreak, the Equateur Province outbreaks, and later smaller outbreaks (23, 26, 27). This pattern does not necessarily reflect a simple failure of EAC governments; rather, it reflects a rational short-term response to constrained budgets, competing political priorities, and historically available external financing.

In 2026, the EAC’s long-standing external funding backstop for Ebola preparedness weakened, lacking a clear replacement plan or a phased transition. The Ebola Bundibugyo outbreak therefore represents the first major EVD test for the EAC under a funding environment in which external support may continue but can no longer be assumed at the historical scale or speed seen in previous outbreaks. This is not an isolated exception but a warning about a broader structural shift in regional health security financing.

The central question is no longer whether EAC health ministries, Africa CDC, and regional political leaders can build more sovereign health-security capacity. It is whether the current crisis will generate concrete budget commitments, institutional permanence, and political accountability. Actionable priorities include protected domestic budget lines for surveillance and IPC, a funded EAC cross-border preparedness mechanism, ongoing funding for community health workers, pooled regional procurement of PPE and diagnostics, in-country laboratory and genomic surveillance capacity, and pre-approved clinical research platforms for vaccines, therapeutics, and survivor follow-up. The DRC experience shows that donor-supported investments can produce durable capacities when they are integrated into national institutions; the policy challenge is to protect those gains while progressively shifting ownership, financing, and accountability to domestic and regional systems.

## Data Availability

The original contributions presented in the study are included in the article/supplementary material, further inquiries can be directed to the corresponding authors.

## Author’s note

Some authors have worked in outbreak preparedness.
